# Anchoring CoO Domains on CoSe_2_ Nanobelts as Bifunctional Electrocatalysts for Overall Water Splitting in Neutral Media

**DOI:** 10.1002/advs.201500426

**Published:** 2016-04-08

**Authors:** Kaidan Li, Jingfang Zhang, Rui Wu, Yifu Yu, Bin Zhang

**Affiliations:** ^1^ Department of ChemistrySchool of Science, and Tianjin Key Laboratory of Molecular Optoelectronic ScienceTianjin UniversityTianjin300072P. R. China; ^2^ Collaborative Innovation Center of Chemical Science and EngineeringTianjin300072P. R. China

**Keywords:** bifunctional electrocatalysts, cobalt, neutral conditions, surface partial oxidation, water splitting

## Abstract

**A facile in situ partial surface‐oxidation strategy to integrate CoO domains with CoSe_2_ nanobelts on Ti mesh** (denoted as CoO/CoSe_2_) via direct calcination of CoSe_2_‐diethylenetriamine precursors is reported. The resulted self‐supported CoO/CoSe_2_ exhibits an outstanding activity and stability in neutral media toward both hydrogen evolution reaction and oxygen evolution reaction.

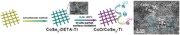

Electrochemical water splitting, including hydrogen evolution reaction (HER) and oxygen evolution reaction (OER), is considered to be a promising technology for sustainable energy conversion, storage, and transport.^1^ Up to now, Pt and RuO_2_ have showed most efficient behavior for HER and OER, respectively.^2^ Unfortunately, the exorbitant price and rarity of noble metals significantly hinder their widespread application. Alternatives based on earth‐abundant elements, such as transition metal carbides,^3^ chalcogenides,^4^ phosphides,^5^ and metal alloys^6^ for HER, as well as oxides,^7^ (oxy)hydroxides,^8^ and phosphates^9^ for OER, have been developed. In spite of their achieved remarkable electrocatalytic behaviors in HER or OER, few active electrocatalysts can function well toward both HER and OER in a same electrolyte because of their unstable or inactive property in unfavorable pH environment.^10^ Therefore, exploring bifunctional catalysts with excellent activity and long‐time stability for both HER and OER has become a hot spot.^11^ Despite some encouraging progress, most bifunctional eletrocatalysts perform in alkaline media,^12^ which impedes their low‐cost scalable deployment in sustainable energy supplies. HER and OER electrocatalysts in neutral media may circumvent many problems generated by electrocatalysts in acidic or alkaline solutions, due to the benign and harmless environment.^13^ It is thus highly imperative but challenging to develop efficient bifunctional catalysts to achieve the overall water splitting under neutral conditions.

Metallic electrocatalysts have sparked great interest owing to their good conductivity and thus remarkable electroactivity.^14^ Although cobalt chalcogenides, typically metallic CoSe_2_,^15^ show extraordinary achievement on catalyzing HER or OER, the low electrochemical performance of pure CoSe_2_ remains to be further improved. One of efficient ways is to form hybrid materials modified with other foreign functional materials.^16^ For instance, Yu et al. reported a novel MoS_2_/CoSe_2_ hybrid with an excellent HER activity in acidic media^17^ and an efficient CeO_2_/CoSe_2_ composite electrocatalyst toward OER in alkaline solutions.^18^ However, the development of metallic‐material‐based bifunctional hybrids for efficient overall water splitting, especially in neutral media, is rarely reported.

Herein, we present a facile in situ partial surface‐oxidation strategy to integrate CoO domains with CoSe_2_ nanobelts on Ti mesh (denoted as CoO/CoSe_2_) as a novel, highly active and stable self‐supported electrocatalyst for both HER and OER under neutral conditions. The strategy can not only avoid the additional increase of nanobelt thickness but also make the hybrid materials to combine tightly. It is believed that the unique 3D self‐supported porous architecture and the chemical synergistic effect between metallic CoSe_2_ and in situ surface oxidized CoO domains thereon lead to the excellent performance.

As illustrated in **Figure** **1**a, the CoO/CoSe_2_ was successfully synthesized by one‐step calcination of CoSe_2_‐DETA (DETA = diethylenetriamine) precursors (Figure S1, Supporting Information) at 450 °C in the mixed O_2_/Ar (0.018 vol% O_2_) atmosphere. The X‐ray diffraction (XRD) pattern (Figure 1b) was first used to reveal the phases of calcined products scraped off from Ti mesh, which corresponds to CoO (JCPDS No. 43‐1004) and CoSe_2_ (JCPDS No. 09‐0234) without any peaks of other impurities. Energy‐dispersive X‐ray (EDX) analysis shows the existence of Co, Se, and O elements, further confirming the formation of CoO/CoSe_2_ (Figure S2, Supporting Information). Figure 1c,d shows scanning electron microscopy (SEM) images of CoO/CoSe_2_, indicating the whole surface of the Ti mesh is decorated with CoO/CoSe_2_ nanobelts with retention of original nanobelt‐like morphology of CoSe_2_‐DETA precursors. These nanobelts have widths of ≈30–300 nm and lengths of several micrometers, which can be bent and interlaced into 3D porous structure for effective electron and mass transfer.^19^ Note that there would be no additional increase of nanobelt thickness related to the exposed active sites^20^ due to the direct calcination strategy without the addition of foreign material. Transmission electron microscopy (TEM) image displays the retained nanobelt structure of CoO/CoSe_2_ (Figure 1e). Typical high resolution TEM (HRTEM) images (Figure 1f and Figure S3, Supporting Information) reveal that small domains (marked with azure dotted areas) with diameters of <7 nm are anchored on the nanobelts. The lattice spacing of 2.10 and 2.60 Å, as expected for CoO (200) planes and CoSe_2_ (210) planes, are observed for domains and nanobelts, respectively. Additionally, high‐angle annular dark field image and the associated scanning transmission electron microscope EDX (STEM‐EDX) element mapping images (Figure 1g) reflect that the Co, Se, and O atoms are distributed over the entire nanobelt. These results confirm that CoO domains, rather than CoO oxidation layer, are successfully anchored on CoSe_2_ nanobelts by such facile in situ partial surface‐oxidation strategy.

**Figure 1:**
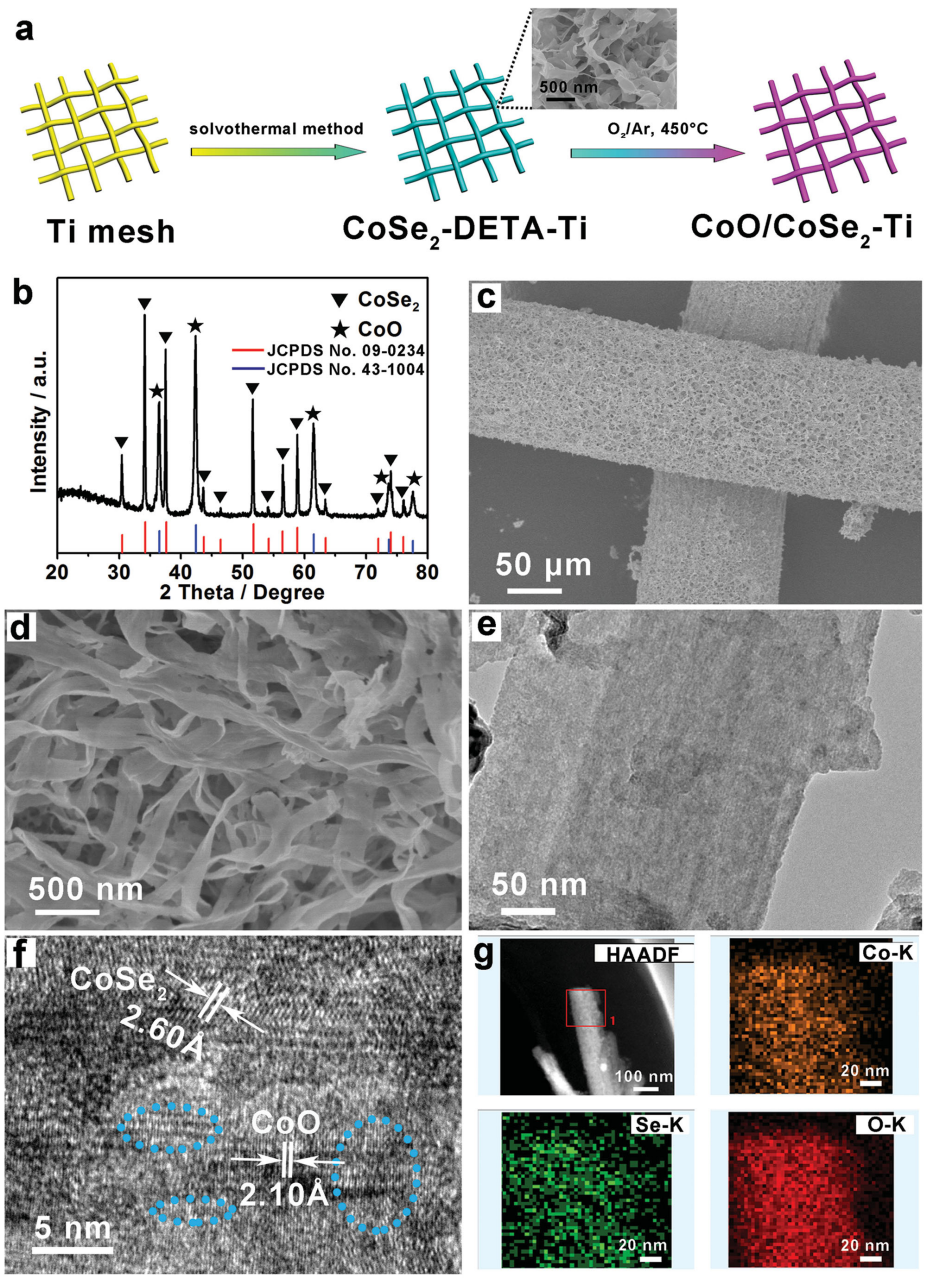
a) Schematic illustration of synthesis of CoO/CoSe_2_. b) XRD pattern of CoO/CoSe_2_. c) Low‐ and d) high‐magnification SEM, e) TEM, f) HRTEM (the azure dotted areas are some CoO domains), and g) STEM‐EDX element mapping images of CoO/CoSe_2_.

The X‐ray photoelectron spectroscopy (XPS) measurements were carried out to unravel the electronic structure. For comparison, pure CoSe_2_ nanobelts were synthesized in high‐purity Ar atmosphere (Figure S4, Supporting Information). **Figure** **2**a shows the Se 3d spectrum of CoO/CoSe_2_, the binding energies at 53.7 and 58.6 eV correspond to Se_2_^2−^ and surface oxidized Se, respectively.^18^ Figure 2b shows the comparison of binding energies of Co 2p in CoO/CoSe_2_ and pure CoSe_2_ nanobelts. The binding energies located at 775–783 eV (Co 2p_3/2_) and 792–798 eV (Co 2p_1/2_) originated from Co^2+^ are observed in pure CoSe_2_ and CoO/CoSe_2_. The two Co 2p peaks and satellite peaks at the higher binding energy side testify the antibonding orbital of Co‐Se and the near‐optimal electronic configuration in connection with electroactivity.^15^ The slight negative shift (≈1 eV) in the binding energy of Co 2p in CoO/CoSe_2_ (779.6 eV) compared with pure CoSe_2_ (780.6 eV) nanobelts suggests the change of electronic structure due to the electron transfer between CoO and CoSe_2_. Meanwhile, the electron donation would make CoO more Lewis acidic and thus activate the H_2_O molecules through Lewis acid–base interaction, which is beneficial for electrocatalytic water splitting.^18^, ^21^

**Figure 2:**
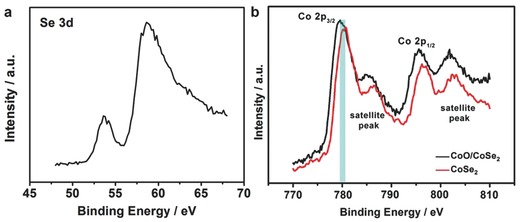
a) XPS spectra of the Se 3d region for CoO/CoSe_2_. b) XPS spectra of the Co 2p region for CoO/CoSe_2_ and pure CoSe_2_.

The electrocatalytic behaviors of CoO/CoSe_2_ as a self‐supported electrode for HER were first tested with a typical three‐electrode system in phosphate buffer solution. For comparison, bare Ti mesh, pure CoSe_2_, pure CoO (Figure S5, Supporting Information), CoO‐CoSe_2_ (physical mixture), and Pt/C (20 wt%, Johnson Matthey) deposited on Ti mesh with the same amount were also measured under the same conditions. **Figure** **3**a shows their *I–R* corrected linear sweep voltammetry (LSV) curves. As expected, the bare Ti mesh has a negligible electrocatalytic activity toward HER and Pt/C shows the best performance with nearly zero overpotential. Surprisingly, the CoO/CoSe_2_ exhibits a large current density with an onset overpotential of 150 mV and a sharply rising current density when a more negative potential is applied, suggesting the high HER activity of CoO/CoSe_2_. And it requires overpotentials of 200 and 337 mV to reach 2 and 10 mA cm^−2^, respectively. Importantly, CoO/CoSe_2_ can achieve a large current density of 50 mA cm^−2^ under a small overpotential of 434 mV. However, pure CoSe_2_, pure CoO, and CoO‐CoSe_2_ (physical mixture) as control catalysts require 430, 538, and 450 mV to obtain 10 mA cm^−2^. These high performances of CoO/CoSe_2_ are comparable or superior to those of some nonprecious metal HER catalysts in neutral conditions, as shown in Table S1 (Supporting Information). The enhanced HER performance may originate from the synergistic effect^22^ of CoO and CoSe_2_. To gain further insights into the HER kinetics, Tafel slopes of CoO/CoSe_2_, pure CoSe_2_, and Pt/C were probed (Figure 3b). For the Pt/C electrocatalyst, a value of 62 mV dec^−1^ is calculated. The measured Tafel slope of CoO/CoSe_2_ is 131 mV dec^−1^ in neutral media. This value is smaller than that of CoSe_2_ (138 mV dec^−1^), demonstrating the faster HER kinetics of CoO/CoSe_2_. Meanwhile, electrochemical impedance spectroscopy (EIS) data of pure CoSe_2_ and CoO/CoSe_2_ were performed at −0.34 V (Figure 3c). The lower charge‐transfer resistance observed for CoO/CoSe_2_ (10 Ω) relative to CoSe_2_ (32 Ω) also suggests its efficient charge transport and thus better electrocatalytic activity.^23^ Furthermore, the exchange current density (*j*_0_) was assessed by extrapolation using the Tafel plot (Figure S6, Supporting Information).[[qv: 5c,24]] The *j*_0_ for CoO/CoSe_2_ is determined to be 33.2 μA cm^−2^ in neutral media, outperforming those of pure CoSe_2_ (6.62 μA cm^−2^) used in this study. To investigate the stability of the CoO/CoSe_2_, a long‐term *I–t* (current density vs time) curve was recorded. Figure 3d shows that the HER performance can be maintained for over 9 h without degradation. Inset of Figure 3d further indicates the excellent electrocatalytic activity remains unchanged after 2000 cycles. These results clearly highlight that CoO/CoSe_2_ is an efficient electrocatalyst with high activity and stability toward HER in neutral media.

**Figure 3:**
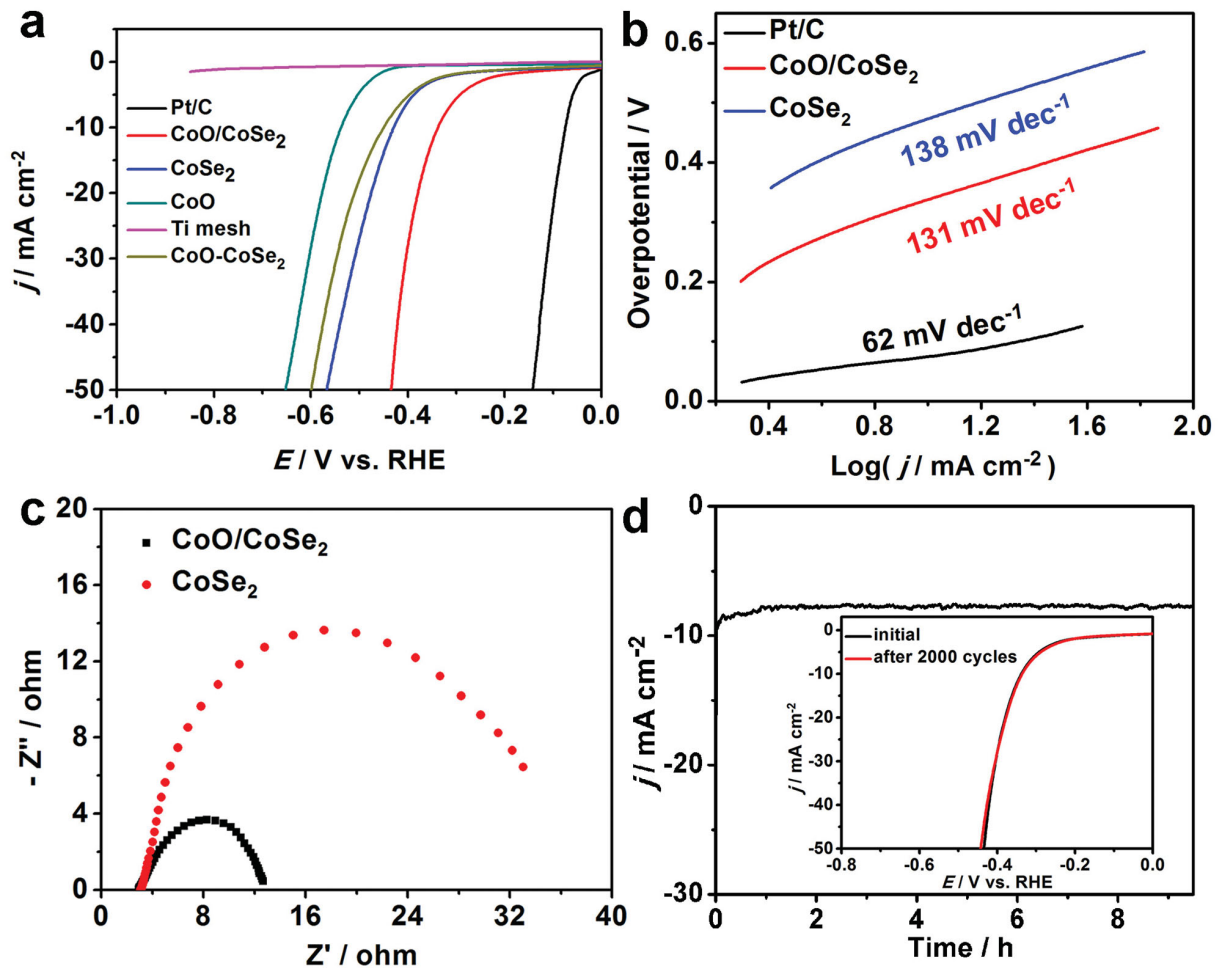
a) LSV curves of bare Ti mesh, pure CoO, pure CoSe_2_, CoO/CoSe_2_, CoO‐CoSe_2_ (physical mixture), and commercial Pt/C (scan rate: 10 mV s^−1^) for HER in 0.5 m phosphate buffer solution (pH = 6.86). b) The corresponding Tafel plots for pure CoSe_2_, CoO/CoSe_2_, and commercial Pt/C. c) EIS of pure CoSe_2_, CoO/CoSe_2_ for HER at −0.34 V. d) *I–t* (current density vs time) curve of CoO/CoSe_2_. Inset is LSV curves of CoO/CoSe_2_ before and after 2000 potential cycles.

The electrocatalytic OER performance for CoO/CoSe_2_ was also examined in phosphate buffer solution. As revealed by **Figure** **4**a, the CoO/CoSe_2_ electrocatalysts show much lower onset overpotential of 320 mV and require an overpotential of 510 mV to achieve 10 mA cm^−2^. In contrast, pure CoSe_2_, pure CoO, CoO‐CoSe_2_ (physical mixture), and commercial RuO_2_ require 650, 610, 630, and 510 mV to obtain 10 mA cm^−2^, respectively. Note that the CoO/CoSe_2_ needed lower overpotentials at large current densities (above 10 mA cm^−2^) compared with commercial RuO_2_. Notably, the current density of CoO/CoSe_2_ at an overpotential of 620 mV is 33.96 mA cm^−2^. The value is 4.5 and 3.1 times than those of pure CoSe_2_ (7.51 mA cm^−2^) and CoO (11.03 mA cm^−2^) and superior to that of commercial RuO_2_ (28.3 mA cm^−2^), respectively, making it one of the most outstanding nonprecious metal OER electrocatalysts in neutral solutions (Table S2, Supporting Information). Also, when an overpotential of 660 mV is applied, CoO/CoSe_2_ can obtain a current density of ≈48 mA cm^−2^, whereas those of pure CoSe_2_ and CoO were below 16 mA cm^−2^. It is worthy to point out that no observation for obvious oxidation peak at 1.1–1.2 V assigned to Co^2+^ to Co^3+^,^25^ implying that the CoO/CoSe_2_ is stable and the excellent activity is from CoO/CoSe_2_ itself under the measurement conditions. The commercial RuO_2_ shows a Tafel slope of 162 mV dec^−1^ (Figure S7, Supporting Information). The Tafel slope for CoO/CoSe_2_ was 137 mV dec^−1^, whereas pure CoSe_2_ and CoO were evaluated as 198 and 183 mV dec^−1^ (Figure 4b). The charge‐transfer resistance for CoO/CoSe_2_ was 12 Ω, which is smaller than that of pure CoSe_2_ (57 Ω) and CoO (42 Ω) (Figure 4c). The smaller Tafel slope as well as lower charge‐transfer resistance demonstrates faster reaction kinetics and the higher electrical conductivity of CoO/CoSe_2_.[[qv: 23c,26]] Figure 4d shows that the catalytic activity of CoO/CoSe_2_ keeps almost unchanged for more than 12 h. After 2000 cycles in phosphate buffer solution, the LSV curve of CoO/CoSe_2_ almost appeared overlapping with the initial curve (Inset of Figure 4d). All the results suggest that CoO/CoSe_2_ exhibits unprecedentedly remarkable activity and stability toward OER in neutral media.

**Figure 4:**
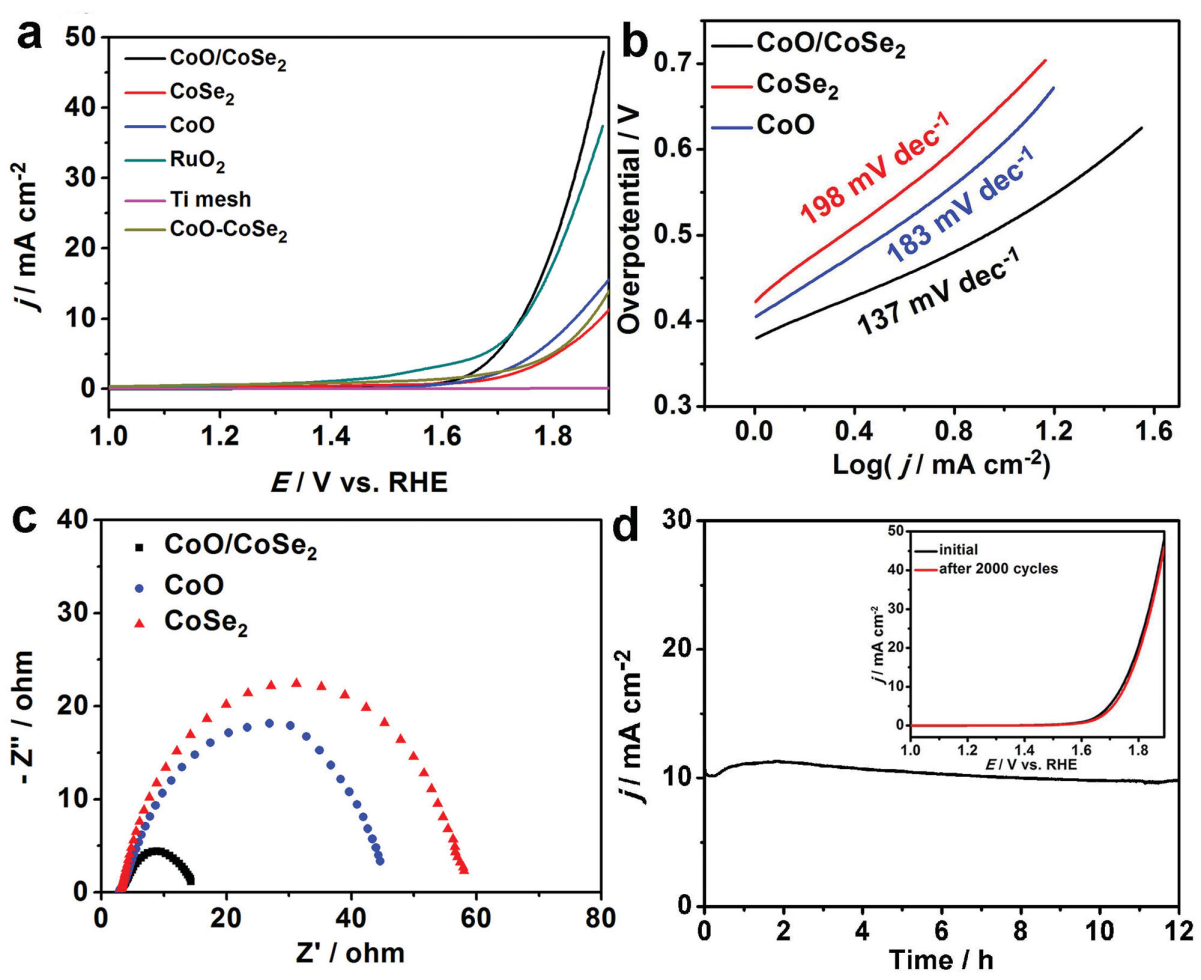
a) LSV curves of bare Ti mesh, pure CoO, pure CoSe_2_, RuO_2_, CoO‐CoSe_2_ (physical mixture), and CoO/CoSe_2_ (scan rate: 10 mV s^−1^) for OER in 0.5 m phosphate buffer solution (pH = 6.86). b) Tafel plots for pure CoSe_2_, pure CoO, and CoO/CoSe_2_. c) EIS of pure CoSe_2_, CoO/CoSe_2_ for OER at 1.75 V. d) *I–t* curve of CoO/CoSe_2_. Inset is LSV curves of CoO/CoSe_2_ before and after 2000 potential cycles.

Moreover, double layer capacitance (*C*_dl_) measurements were conducted to estimate the electrochemical active areas.^27^ CoO/CoSe_2_ shows a *C*_dl_ of 1.8 mF cm^−2^, much higher than that of pure CoSe_2_ (0.38 mF cm^−2^), revealing that the CoO/CoSe_2_ has an advantage in enlarging the active surface area associated with more catalytic active sites than pure CoSe_2_ (**Figure** **5**).

**Figure 5:**
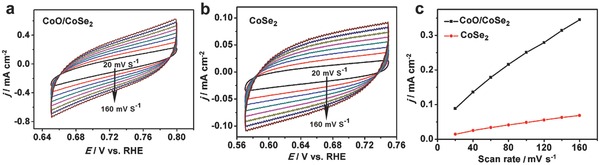
a,b) Cyclic voltammetry (CV) graphs of CoO/CoSe_2_ and pure CoSe_2_ measured at different scan rates from 20 to 160 mV s^−1^. c) Plots of the current density versus the scan rate for CoO/CoSe_2_ and pure CoSe_2_.

To probe the change of morphology and electronic structure occurring for CoO/CoSe_2_ electrocatalyst, we tested SEM, TEM, and XPS data after a series of electrochemical tests. The SEM and TEM images suggest that the CoO/CoSe_2_ electrocatalyst still maintains the original nanobelt structure after HER and OER measurements (Figure S8, Supporting Information). The corresponding XPS spectra of Co 2p region indicate that a similar peak profile is displayed for post‐HER (780.4 eV) or post‐OER (780.2 eV) catalysts in spite of a slight positive shift compared with the as‐prepared CoO/CoSe_2_ electrocatalyst (779.6 eV) (Figure S9, Supporting Information). These results demonstrate that the CoO/CoSe_2_ electrocatalyst still retains the high performance after long‐term HER or OER tests.

With its superior activity and good stability toward both HER and OER in neutral media, we used CoO/CoSe_2_ electrocatalyst as both anode and cathode in a two‐electrode system to make a neutral electrolyzer for water splitting. The electrolyzer allows for 10 mA cm^−2^ in phosphate buffer solution under the applied voltage of 2.18 V (**Figure** **6**a). Although a little more power is needed,^11^, ^28^ the unique CoO/CoSe_2_ as a bifuntional electrocatalyst in neutral media is no less of a breakthrough in water splitting. Additionally, the CoO/CoSe_2_ demonstrates a steady current density of 10 mA cm^−2^ for ≈10 h at a constant voltage of 2.18 V in neutral media (Figure 6b). Furthermore, for overall water splitting, CoO/CoSe_2_ shows ≈100% Faradaic efficiency for both HER and OER with the 1:2 molar ratio of O_2_ and H_2_ in neutral media (Figure 6c).

**Figure 6:**
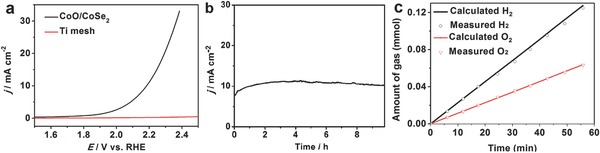
a) LSV curve of water electrolysis employing CoO/CoSe_2_ and Ti mesh as both anode and cathode in 0.5 m phosphate buffer solution. b) *I–t* curve of CoO/CoSe_2_. c) The amount of gas theoretically calculated and experimentally measured versus time for overall water splitting of CoO/CoSe_2_.

In summary, 3D self‐supported CoO/CoSe_2_ electrocatalysts have been successfully synthesized via a facile in situ partial surface‐oxidation method. The materials exhibit superior activity and stability in neutral media for both HER and OER. This excellent performance may be ascribed to two reasons: (1) the flexible nanobelts and their stacking‐growth on Ti mesh endow them with 3D porous architecture, and thus large active surface area, efficient electron, and mass transport; (2) the synergistic effect of metallic CoSe_2_ and in situ oxidized CoO domains in the unique CoO/CoSe_2_ hybrid material yields superactive catalytic sites. Importantly, this cost‐effective in situ chemical transformation approach would open a new avenue to design and explore other novel hybrid materials as efficient catalysts for renewable energy applications.

## Experimental Section

*Synthesis of CoSe_2_/DETA Nanobelts Grown on Ti Mesh*: The CoSe_2_/DETA (DETA = diethylenetriamine) nanobelts grown on Ti mesh were first synthesized by the reported method with some modifications.^29^ First, 186.8 mg of Co(AC)_2_•H_2_O was added into 4 mL of deionized water and then stirred for 5 min to form a pink solution. Simultaneously, 60 mg of NaOH was dissolved into 4 mL of deionized water containing 83.2 mg of SeO_2_. The solutions were mixed and added into 24 mL of DETA solution under constant stirring. A piece of clean Ti mesh (1 × 2 cm^2^, see the SEM image in Figure S10, Supporting Information) was ultrasonicated with acetone, water, and 3.0 m HCl aqueous solution for 10 min, respectively, and then immerged in the above‐mentioned mixed solution. Then, the solution containting Ti mesh was transferred into a 40 mL Teflon‐lined autoclave, kept at 180 °C for 16 h and cooled down naturally to room temperature. The Ti mesh with black precipitates on surface was collected and washed with deionized water and ethanol, and then dried under vacuum at room temperature for 6 h.

*Synthesis of CoO/CoSe_2_ Nanocomposites on Ti Mesh*: In a typical procedure, the Ti mesh with CoSe_2_/DETA on surface was placed in the center of the tube furnace. The furnace was heated to 450 °C with a heating rate of 2 °C min^−1^, and treated at this temperature for 4 h in the O_2_ (0.018 vol%)/Ar atmosphere. The sample was taken out of the furnace after naturally cooled to room temperature.

*Synthesis of Pure CoSe_2_ on Ti Mesh*: For fair comparison, the pure CoSe_2_ was obtained by annealing in the high‐purity Ar atmosphere without detectable O_2_ using Agilent 7890A gas chromatography, with other conditions remaining the same to those of CoO/CoSe_2_ nanocomposites.

*Characterization*: The SEM images and EDX spectroscopic analysis were taken with a Hitachi S‐4800 scanning electron microscope equipped with the Thermo Scientific energy‐dispersion X‐ray fluorescence analyzer. TEM and HRTEM images were obtained with FEITecnai G2 F20 system equipped with GIF 863 Tridiem (Gatan), and EDX elemental distribution images were determined by JEM 2100F transmission electron microscope. Specimens for TEM and HRTEM measurements were prepared via ultrasonicating to strip the samples off the Ti mesh substrate and then dropcasting a droplet of ethanol suspension onto a copper grid and allowed to dry in air. The XRD patterns of the products were recorded with Rigaku D/MAX‐2500 diffractometer (Rigaku Co., JAPAN) using a Cu Kα source (*λ* = 0.154178 nm). XPS analysis was performed on a PHI 5000 Versaprobe system using monochromatic Al Kα radiation. All binding energies were referenced to the C 1s peak at 284.8 eV.

*Electrochemical Measurements*: Electrochemical measurements were carried out in a typical three‐electrode cell consisting of a working electrode, a glassy carbon counter electrode, and a saturated calomel reference electrode (SCE) using an electrochemical workstation (CHI 660D, CH Instruments, Austin, TX). The Ti mesh with CoO/CoSe_2_ catalyst samples directly grown on surface was used as the working electrode. The catalysts as control experiments were dispersed in water/isopropanol with Nafion solution and drop‐cast into the Ti mesh. All the loading mass of the catalysts on the Ti mesh is about 2.0 mg cm^−2^. All the potentials are calibrated to the reversible hydrogen electrode (vs RHE) according to *E*_vs RHE_ = *E*_vs SCE_ + *E*°_SCE_ + 0.059pH and the current density is normalized to the effective geometrical surface area. HER and OER measurements are carried out in the presence of Ar‐saturated phosphate buffer solution (pH = 6.86) as electrolyte. For LSV measurements, the scan rate was set to be 10 mV s^−1^ and all LSV data have been corrected based on *IR* compensation. Comparing the polarization curves before and after *I–R* corrected, it can be seen that *IR* correction has the effect of shifting the raw data to lower potentials under larger current densities (Figure S11, Supporting Information). The continuous cyclic voltammetry (CV) cycling was measured from 0 to −0.4 V versus RHE for HER and from 1.4 to 1.9 V versus RHE for OER with a scan rate of 10 mV s^−1^, respectively. The EIS measurements were carried out in the given overpotential from 100 KHz to 0.1 Hz. CVs in *C*_dl_ determination were measured in a potential window nearly without Faradaic process at different scan rates of 20, 40, 60, 80, 100, 120, 140, and 160 mV s^−1^. The plot of current density at set overpotential against scan rate has a linear relationship and its slope is the *C*_dl_. For full water splitting, we used CoO/CoSe_2_ electrocatalyst as both anode and cathode in a two‐electrode system. The Faradaic efficiency was calculated by comparing the amount of gas theoretically calculated and experimentally measured. To assess the Faradic efficiency, we collected H_2_ and O_2_ by water‐gas displacing method, and calculated the moles of H_2_ and O_2_ generated from the overall water splitting. And then calculate the theoretical amount of H_2_ and O_2_ with *I–t* curve by applying the Faraday law.

## Supporting information

As a service to our authors and readers, this journal provides supporting information supplied by the authors. Such materials are peer reviewed and may be re‐organized for online delivery, but are not copy‐edited or typeset. Technical support issues arising from supporting information (other than missing files) should be addressed to the authors.

Supplementary

## References

[1] a) J. A. Turner , Science 2004, 305, 972; 15310892 10.1126/science.1103197

[2] a) E. A. Paoli , F. Masini , R. Frydendal , D. Deiana , C. Schlaup , M. Malizia , T. W. Hansen , S. Horch , I. E. Stephens , I. Chorkendorff , Chem. Sci. 2015, 6, 190; 10.1039/c4sc02685c PMC5424673 28553467

[3] a) H. Vrubel , X. Hu , Angew. Chem., Int. Ed. 2012, 51, 12703; 10.1002/anie.201207111 23143996

[4] a) J. Yuan , J. Wu , W. J. Hardy , P. Loya , M. Lou , Y. Yang , S. Najmaei , M. Jiang , F. Qin , K. Keyshar , H. Ji , W. Gao , J. Bao , J. Kono , D. Natelson , P. M. Ajayan , J. Lou , Adv. Mater. 2015, 27, 5605; 26293810 10.1002/adma.201502075

[5] a) J. Kibsgaard , T. F. Jaramillo , Angew. Chem., Int. Ed. 2014, 53, 14433; 10.1002/anie.201408222 25359678

[6] Q. Lu , G. S. Hutchings , W. Yu , Y. Zhou , R. V. Forest , R. Tao , J. Rosen , B. T. Yonemoto , Z. Cao , H. Zheng , J. Q. Xiao , F. Jiao , J. G. Chen , Nat. Commun. 2015, 6, 6567. 25910892 10.1038/ncomms7567 PMC4382682

[7] a) J. I. Jung , M. Risch , S. Park , M. G. Kim , G. Nam , H. Y. Jeong , Y. Shao‐Horn , J. Cho , Energy Environ. Sci. 2016, 9, 176;

[8] a) M. S. Burke , M. G. Kast , L. Trotochaud , A. M. Smith , S. W. Boettcher , J. Am. Chem. Soc. 2015, 137, 3638; 25700234 10.1021/jacs.5b00281

[9] a) K. Jin , J. Park , J. Lee , K. D. Yang , G. K. Pradhan , U. Sim , D. Jeong , H. L. Jang , S. Park , D. Kim , N. E. Sung , S. H. Kim , S. Han , K. T. Nam , J. Am. Chem. Soc. 2014, 136, 7435; 24758237 10.1021/ja5026529

[10] a) L. G. Bloor , P. I. Molina , M. D. Symes , L. Cronin , J. Am. Chem. Soc. 2014, 136, 3304; 24499042 10.1021/ja5003197

[11] L. L. Feng , G. Yu , Y. Wu , G. D. Li , H. Li , Y. Sun , T. Asefa , W. Chen , X. Zou , J. Am. Chem. Soc. 2015, 137, 14023. 26352297 10.1021/jacs.5b08186

[12] a) Y. Yang , H. Fei , G. Ruan , J. M. Tour , Adv. Mater. 2015, 27, 3175; 25872881 10.1002/adma.201500894

[13] a) M. Chen , Y. Wu , Y. Han , X. Lin , J. Sun , W. Zhang , R. Cao , ACS Appl. Mater. Interfaces 2015, 7, 21852; 26368828 10.1021/acsami.5b06195

[14] a) X. Long , G. Li , Z. Wang , H. Zhu , T. Zhang , S. Xiao , W. Guo , S. Yang , J. Am. Chem. Soc. 2015, 137, 11900; 26338434 10.1021/jacs.5b07728

[15] a) Y. Liu , H. Cheng , M. Lyu , S. Fan , Q. Liu , W. Zhang , Y. Zhi , C. Wang , C. Xiao , S. Wei , B. Ye , Y. Xie , J. Am. Chem. Soc. 2014, 136, 15670; 25310506 10.1021/ja5085157

[16] a) M. Gong , W. Zhou , M. J. Kenney , R. Kapusta , S. Cowley , Y. Wu , B. Lu , M. C. Lin , D. Y. Wang , J. Yang , B. J. Hwang , H. Dai , Angew. Chem., Int. Ed. 2015, 54, 11989; 10.1002/anie.201504815 26307213

[17] M. R. Gao , J. X. Liang , Y. R. Zheng , Y. F. Xu , J. Jiang , Q. Gao , J. Li , S. H. Yu , Nat. Commun. 2015, 6, 5982. 25585911 10.1038/ncomms6982 PMC4309426

[18] Y. R. Zheng , M. R. Gao , Q. Gao , H. H. Li , J. Xu , Z. Y. Wu , S. H. Yu , Small 2015, 11, 182. 25115699 10.1002/smll.201401423

[19] W. Chen , H. Wang , Y. Li , Y. Liu , J. Sun , S. Lee , J. S. Lee , Y. Cui , ACS Cent. Sci. 2015, 1, 244. 27162978 10.1021/acscentsci.5b00227 PMC4827502

[20] L. Wu , Q. Li , C. H. Wu , H. Zhu , A. Mendoza Garcia , B. Shen , J. Guo , S. Sun , J. Am. Chem. Soc. 2015, 137, 7071. 26018822 10.1021/jacs.5b04142

[21] Y. F. Xu , M. R. Gao , Y. R. Zheng , J. Jiang , S. H. Yu , Angew. Chem., Int. Ed. 2013, 52, 8546. 10.1002/anie.201303495 23843181

[22] a) X. Yan , L. Tian , M. He , X. Chen , Nano Lett. 2015, 15, 6015; 26295904 10.1021/acs.nanolett.5b02205

[23] a) M. Gao , W. Sheng , Z. Zhuang , Q. Fang , S. Gu , J. Jiang , Y. Yan , J. Am. Chem. Soc. 2014, 136, 7077; 24761994 10.1021/ja502128j

[24] L. Yu , B. Y. Xia , X. Wang , X. W. Lou , Adv. Mater. 2016, 28, 92. 26539882 10.1002/adma.201504024

[25] Y. Wang , T. Zhou , K. Jiang , P. Da , Z. Peng , J. Tang , B. Kong , W. B. Cai , Z. Yang , G. Zheng , Adv. Energy Mater. 2014, 4, 1400696.

[26] H. J. Qiu , Y. Ito , W. Cong , Y. Tan , P. Liu , A. Hirata , T. Fujita , Z. Tang , M. Chen , Angew. Chem., Int. Ed. 2015, 54, 14031. 10.1002/anie.201507381 26474177

[27] T. Y. Ma , S. Dai , M. Jaroniec , S. Z. Qiao , J. Am. Chem. Soc. 2014, 136, 13925. 25216300 10.1021/ja5082553

[28] C. Tang , N. Cheng , Z. Pu , W. Xing , X. Sun , Angew. Chem., Int. Ed. 2015, 54, 9351. 10.1002/anie.201503407 26136347

[29] M. R. Gao , W. T. Yao , H. B. Yao , S. H. Yu , J. Am. Chem. Soc. 2009, 131, 7486. 19445509 10.1021/ja900506x

