# Supplementary material for: Anchoring CoO Domains on CoSe2 Nanobelts as Bifunctional Electrocatalysts for Overall Water Splitting in Neutral Media
Source: Adv Sci (Weinh). 2016 Apr 8;3(6):1500426. doi: 10.1002/advs.201500426 (PMC5071745; doi:10.1002/advs.201500426)
Supplement: Supplementary file 1 — Supplementary [file ADVS-3-0g-s001.pdf]

Copyright WILEY-VCH Verlag GmbH & Co. KGaA, 69469 Weinheim, Germany, 2016.

ADVANCED SCIENCE Open Access

Supporting Information

for *Adv. Sci.,* DOI: 10.1002/advs.201500426

Anchoring CoO Domains on $CoSe_2$ Nanobelts as Bifunctional Electrocatalysts for Overall Water Splitting in Neutral Media

*Kaidan Li, Jingfang Zhang, Rui Wu, Yifu Yu, and Bin Zhang**

Copyright WILEY-VCH Verlag GmbH & Co. KGaA, 69469 Weinheim, Germany, 2013.

Supporting Information

Anchoring CoO Domains on $CoSe_2$ Nanobelts as Bifunctional Electrocatalysts for Overall Water Splitting in Neutral Media

*Kaidan Li, Jingfang Zhang, Rui Wu, Yifu Yu, and Bin Zhang**

K. Li, J. Zhang, R. Wu, Dr. Y. Yu, Prof. B. Zhang
Department of Chemistry, School of Science, and Tianjin Key Laboratory of Molecular Optoelectronic Science
Tianjin University
Tianjin, 300072, China
E-mail: bzhang@tju.edu.cn

K. Li, J. Zhang, Prof. B. Zhang
Collaborative Innovation Center of Chemical Science and Engineering
Tianjin 300072, China

Characterization of $CoSe_2$-DETA

The $CoSe_2$/DETA (DETA = diethylenetriamine) nanobelts grown on Ti mesh were synthesized by the reported method with slight modifications.[1] We can see from Figure S1, that nanobelts were grown on Ti mesh on a large scale, with 30-300 nm in width and several dozens of micrometers in length. The TEM image further indicates the flexible nanobelt structure. The XRD patterns are consisted with that of the reported $CoSe_2$/DETA,[2] further supporting the formation of $CoSe_2$-DETA precursors.

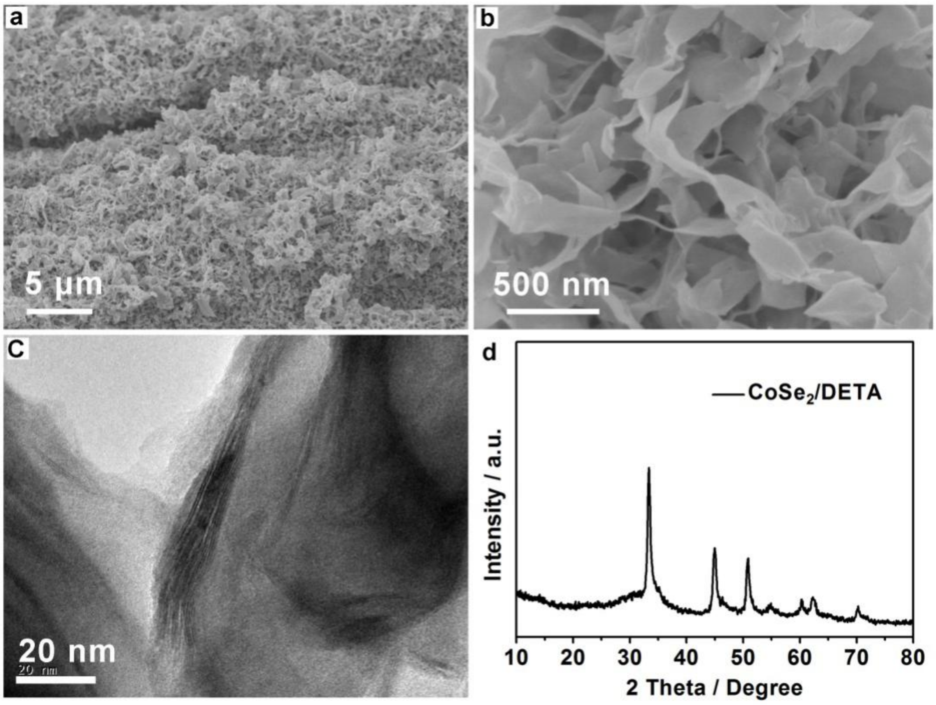

Figure S1. (a) Low- and (b) high-magnification SEM images of $CoSe_2$-DETA precursors. (c) A typical TEM image and (d) XRD pattern of $CoSe_2$-DETA precursors.

Elements in the as-prepared $CoO/CoSe_2$

Energy-dispersive X-ray spectroscopy (EDX) reveals that the sample on Ti mesh is composed of O, Co and Se elements.

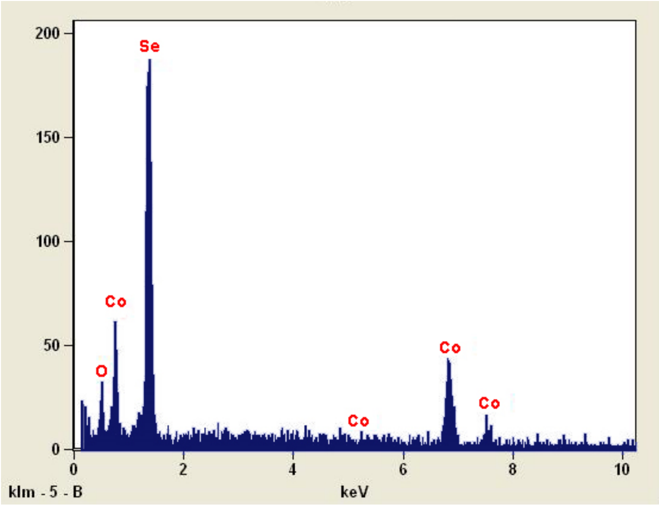

Figure S2. The EDX spectrum of the as-prepared $CoO/CoSe_2$ synthesized by one-step calcination of $CoSe_2$-DETA (DETA = diethylenetriamine) precursors at 450 °C in mixed O_2 (0.018 vol%)/Ar atmosphere.

HRTEM image of $CoO/CoSe_2$

The lattice spacing of 2.10 Å and 2.60 Å, as expected for CoO (200) planes and $CoSe_2$ (210) planes, are observed for domains and nanobelts, respectively. This observation reveals that small CoO domains were anchored on the $CoSe_2$ nanobelts.

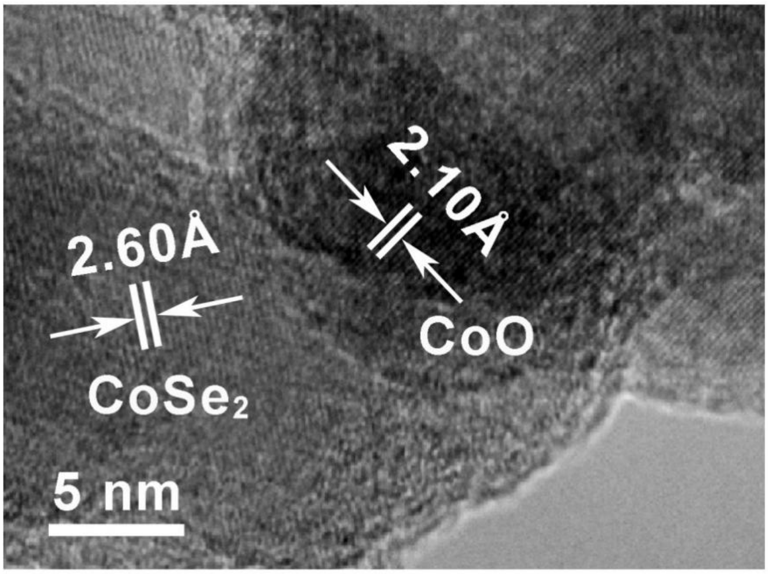

Figure S3. HRTEM image of the as-prepared $CoO/CoSe_2$ synthesized by one-step calcination of $CoSe_2$-DETA (DETA = diethylenetriamine) precursors at 450 °C in mixed O_2 (0.018 vol%)/Ar atmosphere.

Characterization of pure $CoSe_2$

For comparison, the pure $CoSe_2$ was obtained by annealing $CoSe_2$-DETA precursors in high-purity Ar atmosphere with other conditions remaining unchanged. As we can see from Figure S4, nanobelt-like morphology can be preserved, which is similar to that of the as-prepared $CoO/CoSe_2$. Addtionally, all XRD patterns can be attributed to pure $CoSe_2$ (JCPDS no. 09-0234), suggesting that $CoSe_2$ can be synthesized in the absence of oxygen in the Ar atmosphere.

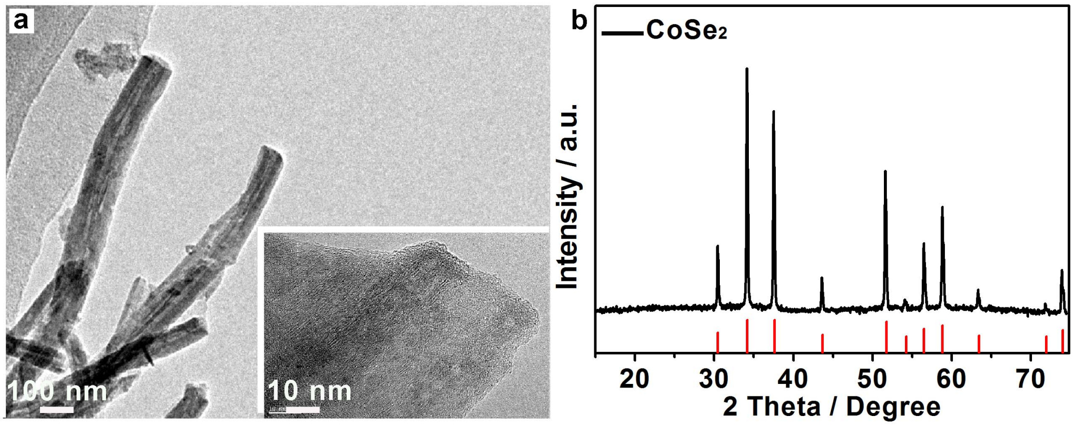

Figure S4. (a) TEM image and (b) XRD pattern of pure $CoSe_2$ nanobelts. Inset in (a) is the high-resolution TEM image.

Characterization of commercial CoO

CoO was purchased from Aladdin Industrial Corporation (AR) and was used as received without further treatment.

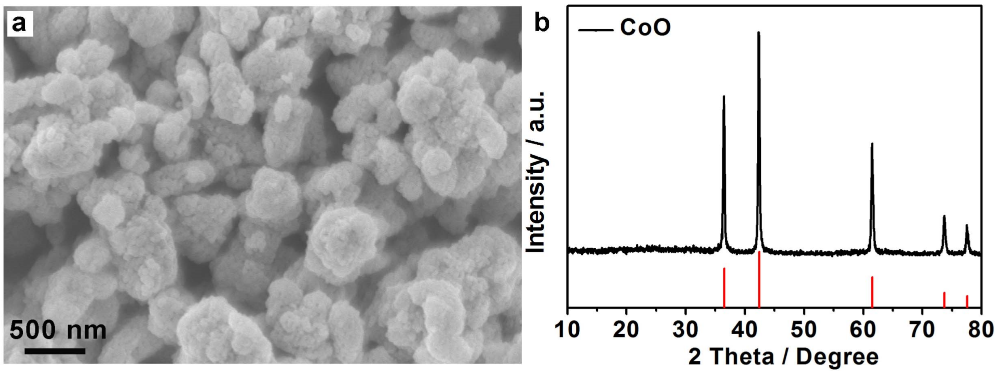

Figure S5. (a) SEM image of CoO. (b) XRD pattern of pure CoO sample corresponding to CoO (JCPDS no. 43-1004).

Calculation of exchange current density

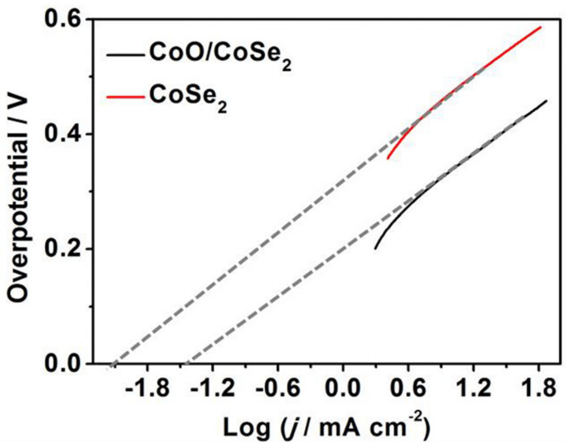

Figure S6. Calculated exchange current density by extrapolating the Tafel plot.

Characterization of commercial RuO_2

RuO_2 was purchased from Aladdin Industrial Corporation (AR) and was used as received without further treatment. The measured Tafel slope of commercial RuO_2 is 162 mV dec^{-1}.

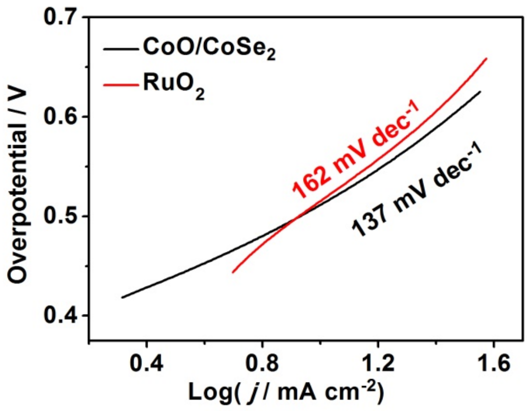

Figure S7. Tafel plots for $CoO/CoSe_2$ and commercial RuO_2.

Stability of $CoO/CoSe_2$ during HER and OER

The SEM and TEM images suggest that the $CoO/CoSe_2$ electrocatalyst still maintains the original nanobelt structure after HER and OER measurements.

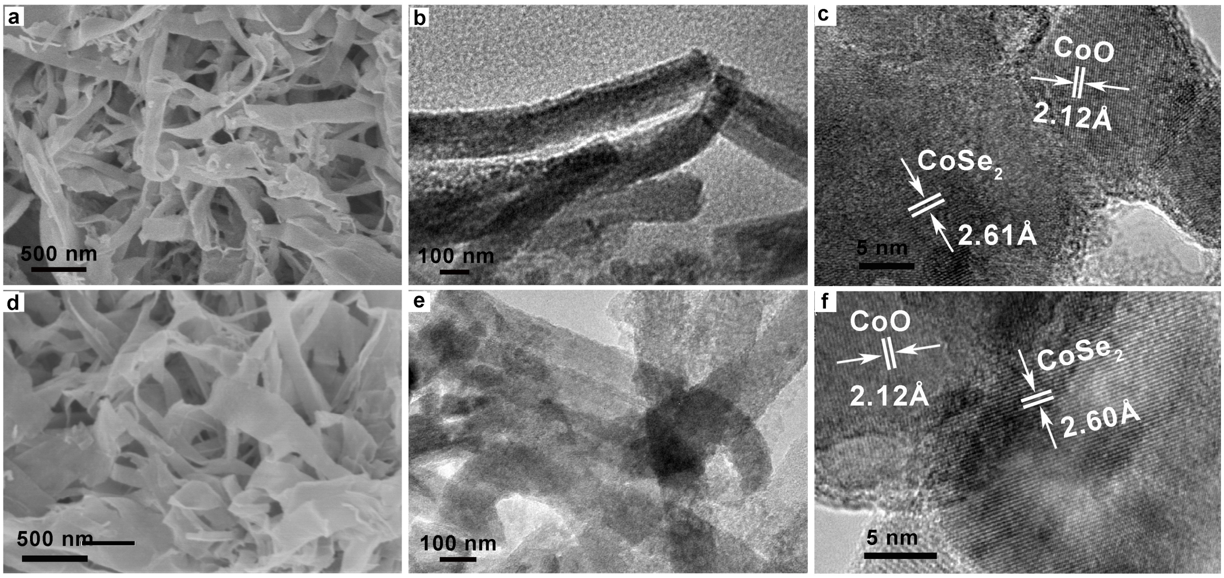

Figure S8. (a) SEM, (b) TEM and (c) HRTEM images of $CoO/CoSe_2$ after HER tests. (d) SEM, (e) TEM and (f) HRTEM images of $CoO/CoSe_2$ after OER tests.

The corresponding XPS spectra of Co 2p region indicate that a similar peak profile is displayed for post-HER (780.4 eV) or post-OER (780.2 eV) catalyst in spite of a slight positive shift compared with the as-prepared $CoO/CoSe_2$ electrocatalyst (779.6 eV).

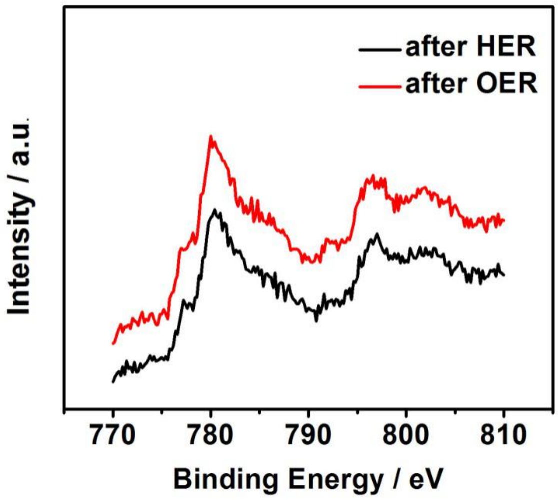

Figure S9. XPS spectra of the Co 2p region for $CoO/CoSe_2$ after HER and OER.

Characterization of Ti mesh

Ti mesh (diameter: 0.12 mm) was purchased from Hongyun Metal Products Co., Ltd.

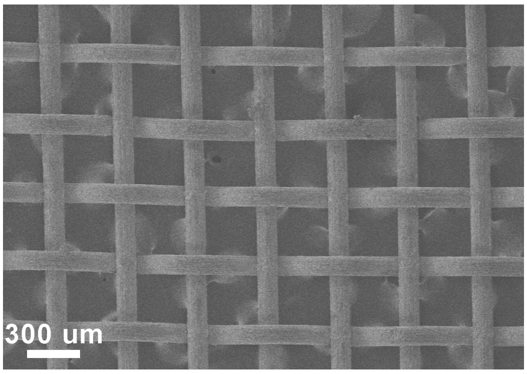

Figure S10. SEM image of Ti mesh.

Linear sweep voltammetry (LSV) curves (HER and OER) before and after I-R corrected

All the potential values measured were calibrated by using the following equation: E_{after}=E_{before}-IR, where I was the current flowing through the cell and R was the ohmic resistance of the cell. The resistances, R, were measured by the IR compensation function available on the electrochemical workstation (CHI 660D, CH Instruments, Austin, TX).

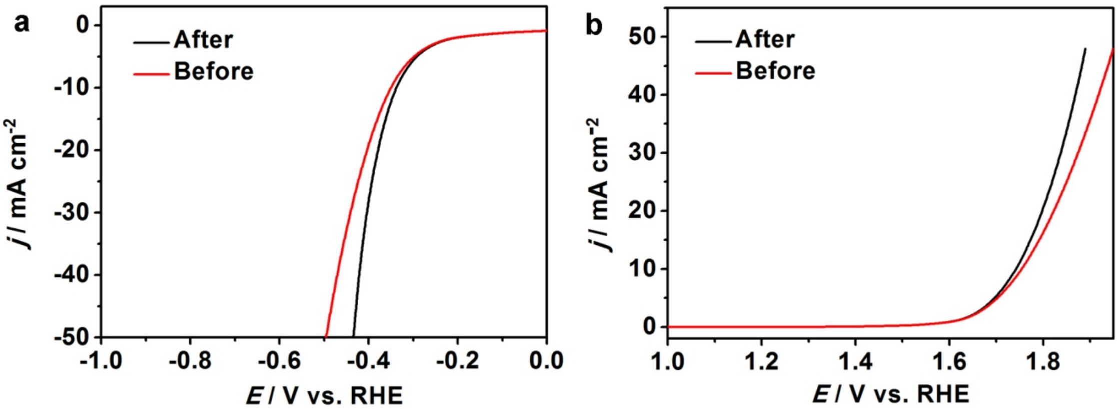

Figure S11. (a) HER polarization curves of $CoO/CoSe_2$ before and after I-R correction. (b) OER polarization curves of $CoO/CoSe_2$ before and after I-R correction.

Table S1. Comparison of the HER activity of $CoO/CoSe_2$ under neutral conditions with some representative HER catalysts recently reported.

Sample	Loading/substrate	electrolyte	Current density (*j*)/ mA cm^{-2}	Overpotential/mV at the corresponding *j*	Reference
Co@nitrogen-rich carbon nanotubes	0.28 mg/cm^2	0.1 M phosphate buffer (pH = 7)	2<br>10	380<br>540	*Angew. Chem.* **2014**, *126*, 4461
$CoO/CoSe_2$	2 mg cm^{-2}<br>Ti mesh	0.5 M phosphate buffer solution (pH = 6.86)	2<br>10<br>50	200<br>337<br>434	This work
Metallic cobalt @ cobalt-oxo / hydroxo phosphate	FTO	0.5M phosphate buffer (KPi, pH = 7)	2	385	*Nat. Mater.* **2012**, *11*, 802
Electrodeposited cobalt-sulfide	Co = 1.35 □μmol/cm^2 (79.6 μg/cm^2) FTO	1.0 M potassium phosphate buffer pH = 7	2	85	*J. Am. Chem. Soc.* **2013**, *135*, 17699
Co–Mo–S film	glassy carbon	phosphate buffer pH = 7	1.04	200	*Chem. Sci.* **2012**, *3*, 2515
Ni_3S_2 nanosheet arrays / Ni foam	1.6 mg/cm^2<br>Ni foam	phosphate buffer pH = 7	10	170	*J. Am. Chem. Soc.* **2015**, DOI: 10.1021/jacs.5b08186
Mo_2C	—	phosphate buffer pH = 7	1	200	*Angew. Chem. Int. Ed.* **2012**, *51*, 12703
Mo_2B	—	phosphate buffer pH = 7	1	250	*Angew. Chem. Int. Ed.* **2012**, *51*, 12703
CoP/CC	0.92 mg cm^{-2}<br>carbon cloth	1.0 M phosphate buffer solution pH = 7	2<br>10	65<br>106	*J. Am. Chem. Soc.* **2014**, *136*, 7587

Table S2. Comparison of the OER activity of $CoO/CoSe_2$ under neutral conditions with some representative OER catalysts recently reported.

Sample	Loading/ substrate	electrolyte	Onset overpotential /mV	Current density (*j*)/ mA cm^{-2}	Overpotential/ mV at the corresponding *j*	Tafel slope /mV dec^{-1}	Reference
Co_3S_4 Nanosheets	0.28 mg/cm^2	phosphate buffer solution (pH = 6.86)	310	3.97	700	151	*Angew. Chem. Int. Ed.* **2015**, *54*, 11231
$CoO/CoSe_2$	2 mg cm^{-2} Ti mesh	0.5 M phosphate buffer solution (pH = 6.86)	320	33.96	620	137	This work
Co-based phosphate/ITO	ITO	0.1 M potassium phosphate (pH = 7.0)	280	1	410	—	*Science*, **2008**, *321*, 1072
$Mn_3(PO_4)_2\cdot 3H_2O$/FTO	FTO	0.5M phosphate buffer (pH = 7)	450	0.316	680	120	*J. Am. Chem. Soc.* **2014**, *136*, 7435
$LiCoPO_4$	0.10 mg/cm^2	0.1 M potassium phosphate (pH = 7.0)	370	—	—	120	*J. Am. Chem. Soc.* **2012**, *134*, 16959
$LiMnP_2O_7$	0.25 mg/cm^2 FTO	phosphate buffer (pH = 7.0)	450	0.5	680	120	*J. Am. Chem. Soc.* **2014**, *136*, *4201*
Ni	17 nM FTO	0.5 M potassium borate (pH = 9.2)	—	1	540	—	*J. Am. Chem. Soc.* **2015**, *137*, 13980

References:

[1] M. R. Gao, W. T. Yao, H. B. Yao, S. H. Yu, *J. Am. Chem. Soc.* **2009**, *131*, 7486.

[2] Y. R. Zheng, M. R. Gao, Q. Gao, H. H. Li, J. Xu, Z. Y. Wu, S. H. Yu, *Small* **2015**, *11*, 182.
